# Biomarkers for Early Complications of Endothelial Origin After Allogeneic Hematopoietic Stem Cell Transplantation: Do They Have a Potential Clinical Role?

**DOI:** 10.3389/fimmu.2021.641427

**Published:** 2021-05-19

**Authors:** Giuseppe Lia, Luisa Giaccone, Sarah Leone, Benedetto Bruno

**Affiliations:** ^1^ Stem Cell Transplant Program, Department of Oncology, A.O.U. Città della Salute e della Scienza di Torino, Torino, Italy; ^2^ Department of Molecular Biotechnology and Health Sciences, University of Torino, Torino, Italy; ^3^ Department of Internal Medicine, New York University Grossman School of Medicine, New York, NY, United States; ^4^ Division of Hematology and Medical Oncology, New York University Grossman School of Medicine, Perlmutter Cancer Center, New York University Langone Health, New York, NY, United States

**Keywords:** HCT, endothelial dysfunction, SOS, GvHD, TA-TMA, biomarkers

## Abstract

Endothelial cell (EC) dysfunction causes a number of early and life-threatening post hematopoietic stem cell transplant (HCT) complications that result in a rapid clinical decline. The main early complications are graft-*vs*.-host disease (GVHD), transplant associated thrombotic microangiopathy (TA-TMA), and sinusoidal obstruction syndrome (SOS). Post-HCT endothelial dysfunction occurs as a result of chemotherapy, infections, and allogeneic reactivity. Despite major advances in transplant immunology and improvements in supportive care medicine, these complications represent a major obstacle for successful HCT. In recent years, different biomarkers have been investigated for early detection of post-transplant endothelial cell dysfunction, but few have been validated. In this review we will define GVHD, TA-TMA and SOS, summarize the current data available in HCT biomarker research and identify promising biomarkers for detection and diagnosis of early HCT complications.

## Introduction

Hematopoietic cell transplantation (HCT) is indicated in a broad range of diseases, most frequently acute leukaemia and myelodysplastic syndromes (1). Despite the advances in hematopoietic stem cell transplants, it remains a high risk procedure that is limited by potentially life-threatening complications (2).

Early (or short-term) post-HCT complications typically occurred within the first 100 days post-HCT. Most complications (e.g. mucositis and sepsis) are caused by infections. They derived from toxicity of the chemotherapy conditioning regimen, which leads to the post-HCT neutropenic phase and the passage of pathogenic bacteria through damaged epithelium and mucosa. Other early complications are not infection-related but equally potentially detrimental. They include engraftment rejection/failure, organ toxicity, acute graft-versus-host disease (GVHD), and other relatively rare complications. These latter group of complications includes sinusoidal obstruction syndrome (SOS), capillary leak syndrome, engraftment and peri-engraftment syndrome, diffuse alveolar haemorrhage, and transplant associated thrombotic microangiopathy (TA-TMA) (3–7).

Recent studies show that endothelial activation and damage are crucial not only for pathogenesis of these latter complications but is also the origin and not the consequence of acute graft-versus-host disease (GVHD). Thus, they are now commonly known as early post-HCT complications of endothelial origin. In addition to early onset time, the post-HCT complications of endothelial origin share a series of common characteristics. They all start at the capillary level (systemically or in one or more organs); furthermore, they are syndromes with overlapping clinical signs and symptoms. Currently, clinical criteria for diagnosis are still not well defined and therefore diagnosing these complications is often tricky due to the lack of specific tests. Additionally, these complications can easily evolve into multiple organ failure (MOF), especially if not promptly recognized. Among early post-HCT complications of endothelial origin, GVHD is the most common and is associated with high rate of morbidity and mortality (8, 9).

Furthermore, endothelial cells (EC) are also involved in regulating and supporting haematopoiesis in the bone marrow micro-environment. EC dysfunction has been linked to delayed hematopoietic recovery, poor graft function and thrombocytopenia after allogeneic HCT (allo-HCT) (10). Moreover, preventing use of drugs with antioxidant and anti-inflammatory activities, like N-acetyl-L-cysteine and atorvastatin, has been correlated with a reduction of endothelial dysfunction and a reduced incidence of poor graft function in patients (11, 12).

**Figure 1:**
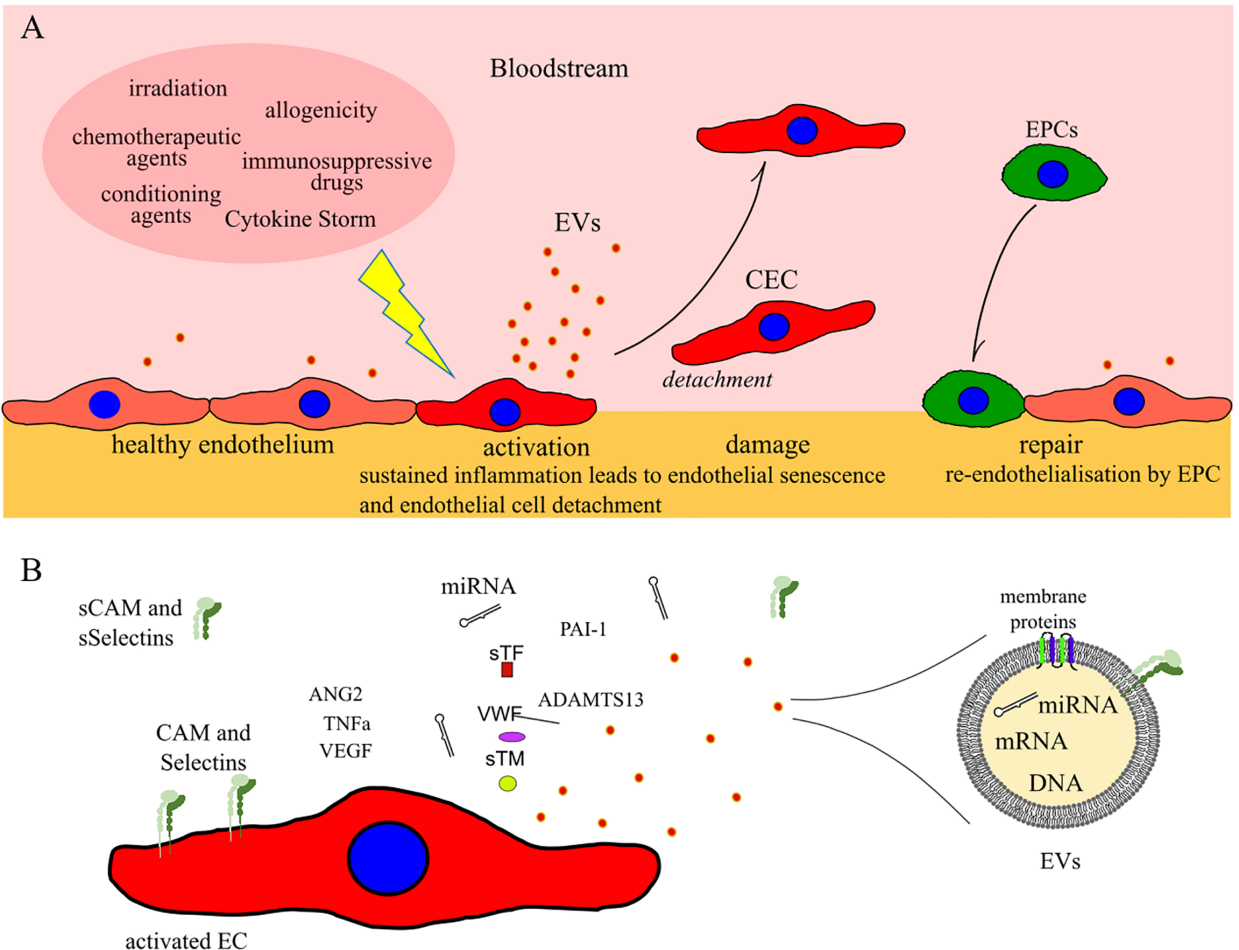
**(A)** Schematic illustrating ECs activation, damage and repair after allogeneic HCT. **(B)** Common markers of EC damage which can potentially be used for detection of early post-HCT complications of endothelial origin. CEC: circulating endothelial cells; EPCs: progenitor endothelial cells; EVs: extracellular vesicles; CAM and sCAM: cell adhesion molecules and soluble CAM; sSelectins: soluble Selectins.

Due to their ubiquitous distribution endothelial cells (EC) are exposed to both physiologic and pathologic stimuli. For example, proinflammatory cytokines and other immunomodulatory agents transform the endothelial environment to a proinflammatory state characterized by reduced vasodilation and prothrombotic properties. This phenomenon, known as EC activation, plays main role in the inflammatory response (**Figure 1**). EC activation can revert back to its pre-inflammatory state or it can led to irreversible dysfunctional state (13). Endothelial dysfunction results in increased leukocytes adhesion and passage through vessel wall, platelet activation, and cytokine release. Together, they generate a vicious circle mechanism that damages the endothelial microenvironment, leading to endothelial cell apoptosis.

Carreras and colleagues demonstrated, in both autologous and allo-HCT, a significant endothelial damage and activation, even in the absence of associated complications (14, 15). Endothelial dysfunction can present very differently after HCT based on when and where the dysfunction occurs (see paragraphs above). The major source of endothelial damage is chemo-radiotherapy used in conditioning regimens (14, 16). EC dysfunction is also promoted by other factors, such as cytokines release (17), bacterial lipopolysaccharides translocated through the damaged mucosal barriers (18), some drugs employed in HCT (e.g. granulocytes-colony-stimulating factor or calcineurin inhibitors) (19, 20), engraftment and allogeneic reactions with the donor-derived immune cells (15, 21).

Endothelial damage and dysregulation of new blood-vessel formation (neovascularization) are risk factors for developing these early HCT complications Therefore, identifying endothelial biomarkers for early detection of EC dysfunction could lead to early treatment and prevention of irreversible EC damage.

Most biomarker studies focus on the most common HCT complications including GVHD, TA-TMA and SOS. In this review, we describe the current biomarker research and highlight potential biomarkers that could have a potential role in clinical practise.

### Acute Graft Versus Host Disease

GVHD is a complex immune reaction that occurs when activated donor T lymphocytes mount a destructive immune response against host tissue. Main acute GVHD organ targets are gastrointestinal (GI) tract, liver and skin. Common clinical presentations of acute GVHD include vomit and watery diarrhea, cholestasis and hyperbiliruminemia, and maculopapular rash, respectively. Between 30-50% of patients who undergo allo-HCTs develop GVHD within 100 days post-HCT (1). Some identified risk factors for GVHD are: presence of Human Leukocyte Antigens (HLA) disparity, older age and gender disparity in donors and recipients, high dose of total-body irradiation, type of GVHD prophylaxis and prior donor alloimmunization (22).

Murine models have largely contributed to this field of study and have elucidated the pathogenesis of GVHD. It is now widely accepted that GVHD develops in three sequential phases. The first phase is characterized by chemotherapy induced Antigen Presenting Cell (APC) activation. The conditioning chemo regimen triggers tissue damage and endothelial cell injury causing arteritis and loss of microvessels (23). During the first phase neutrophils, monocytes and inflammatory cells produce reactive oxygen species as a consequence of tissue damage caused by chemo/radiotherapy and eventual infections, infiltrating the GI tract. Moreover, damage- and pathogen-associated molecular pattern molecules are released from damaged tissues and activate both innate and adaptive immune responses (24). The second phase is characterized by APC activation of donor alloreactive T cells, which migrate to target tissues. In the last phase we observe an increase of Fas-ligand expression and secretion of granzyme B and perforin which mediate targeted patient cell death (25). Furthermore, a massive release of inflammatory cytokines such as interleukin (IL) 1 and 6, interferon gamma (IFNγ), and tumor necrosis factor alpha (TNFα), plays an important role in GVHD pathophysiology and exerts cytotoxic effects on host-cell (26).

There are a number of studies using mouse models and human tissue models that confirm the theory that donor T cells are the driving force of alloreactivity. *In vivo* studies conducted by Biedermann et al. demonstrated that allogeneic reactions against ECs are associated with loss of dermal vessels and presence of CD8+ T cell infiltrates in the skin (27, 28). Moreover, endothelial damage in patients has been correlated with pathogenesis of steroid resistant GVHD and increased non-relapse mortality (NMR) (29). Cordes et al. showed that sildenafil, a phosphodiesterase type 5 inhibitor known to protects EC by improving metabolic activity and reducing apoptosis decreased GVHD effects when given in combination with steroids in experimental models. Additionally, sildenafil improved animal survival reducing EC damage in liver and fibrinogen deposits in colon (30).

Studies conducted on human umbilical vein endothelial cell cultures (EC *in vitro* model) showed that alloreactivity and chemotherapy agents can induce endothelial apoptosis (21). In addition, EC injury and/or dysfunction can also be aggravated by the presence of lipopolysaccharide (LPS) or endotoxin (31). Moreover, preclinical mouse models and clinical observations have shown that markers of neovascularization and endothelial damage are associated with the occurrence of GVHD (32, 33). Furthermore, the endothelium has not only a role in the pathogenesis of GVHD, but being located between the alloreactive donor T-cells and the host’s tissue, is also a main exposed target for direct or indirect immune-mediated injury from either the cytotoxic T-lymphocytes or from the cytokines storm.

The complex interrelationship between endothelial damage and GVHD has led to the development of a new term called ‘endothelial GVHD’; a precursor to the clinical presentation of acute GVHD (34–36). The active involvement of EC in the pathobiology of GVHD is supported in murine GVHD model where the inhibition of neovascularization has led to amelioration of GVHD symptoms and reduced mortality (32, 37). Unfortunately, at the moment very few treatments for damaged endothelium are available for routine clinical use. Interestingly, some studies shown that, in both adults and paediatric patients, the use in prophylaxis of defibrotide (DF), an approved endothelial protective drug for the treatment of SOS (38), accidentally appeared to reduce also the incidence GVHD (39–41). The reduction of GVHD promoted by DF was also confirmed by an *in vivo* study on mouse model (42). A phase two clinical trial (NCT03339297) is now testing whether the use in prophylaxis of DF prevents the development of GVHD.

### Thrombotic Microangiopathy Associated With HCT

Transplant associated thrombotic microangiopathies (TA-TMA) are characterized by an abnormal platelet activation. This results in micro-thrombi deposition and subsequent microangiopathic hemolytic anemia. The accumulation of micro-thrombi also occludes and disrupts the microcirculation, leading to ischemic organ dysfunction, especially renal dysfunction, and neurological abnormalities. Time of onset of TA-TMA is 3 months after HCT and occurs more frequently in allo-HCT, where is typically associated to treatment with calcineurin inhibitors, compared to autologous HCT (5–15% of patients *vs <*1%, respectively) (22). The collection of signs and/or symptoms associated with TA-TMA are highly variables and they range from asymptomatic anemia and low platelets levels to mild acute renal failure and fulminant MOF.

The pathophysiology of TA-TMA is not completely well understood, although endothelial damage appears to have a crucial role in TA-TMA development. Additionally, damaged and dysfunctional EC have a procoagulant activity through exposure of collagen, negative charges, and Tissue Factor which lead to intravascular complement and platelets activation. Aberrant complement activation is dependent on several intrinsic and extrinsic risk factors. Intrinsic risk factors are donor specific and include female sex, age, and genetic predispositions (HLA-mismatch); extrinsic risk factors are modifiable and include conditioning regimen, viral infections, immunosuppressive therapies and GVHD (7).

Endothelial injury with aberrant complement activation can be described by a ‘two-hit’ process. The first “hit” makes the endothelium more procoagulant. This damage occurs in the early post-HCT aplastic phase and is generally caused by conditioning regimen in presence of concurrent risk factors (e.g. prolonged immobilization, severe infections, high dose busulfan) (43). The “second hit” causes further endothelial injury and initiates platelet aggregation and thrombus formation in microvessels. The initiating agents involved in the second “hit” phase are the use of calcineurin inhibitors, especially in association with mTOR inhibitors, the presence of GVHD and/or infections. Furthermore, some data shows that neutrophil extracellular traps (NETs) could also participate in EC damage and in complement activation. Thus, serum NETs levels are significantly elevated in the first weeks after allo-HCT in TA-TMA patients and could be used as a predictive biomarkers of thrombotic microangiopathy (44).

At present, there is not consensus on the diagnostic criteria for TA-TMA, other than histologic examination of the damaged tissues. Furthermore, performing a tissue biopsy for histologic diagnosis of TA-TMA is associated with potential complications in thrombocytopenic patients and cannot be considered a routine procedure. The lack of consistent non-invasive diagnostic criteria is responsible for delayed diagnosis and irreversible organ damage; therefore mortality rate in affected patients is up to 75% within 3 months from TA-TMA onset (45). Since diagnosis of TA-TMA continues to be challenging, new diagnostic criteria set has been proposed (46). A first set was proposed by the European Group for Blood and Bone Marrow Transplantation and the European Leukemia Net International Working Group. It is based on the following 5 criteria: 1) increased schistocytes; 2) new onset or worsening of thrombocytopenia 3) increased Lactate Dehydrogenase; 4) decreased haptoglobin; 5) and decreased haemoglobin concentration or increased red cell transfusion (47). The second set of criteria, proposed by the Blood and Marrow Transplant Clinical Trials Network, includes: number of schistocytes counted with optical microscopy only; Lactate Dehydrogenase; negative Coombs test; and renal dysfunction or unexplained neurological impairment (48).

### Sinusoidal Obstruction Syndrome

Damage of sinusoidal and hepatic venule endothelial cell is crucial for the pathogenesis of SOS. The injury leads to platelet and fibrin deposition in the sub endothelium and fibrosis of the terminal venous venules and sinusoids. These events result in vascular occlusion and subsequent decreased hepatic venous outflow, post-sinusoidal hypertension and eventually hepatocellular necrosis and liver failure (49).

Clinically it is characterized by icterus, fluid retention, and painful enlarged liver that occurs within the first 21-40 days post-HCT (50, 51). Cases presenting within the first 21 days are referred to as classical SOS while those presenting after day 40 are referred to as late onset SOS. Currently, SOS is diagnosed based on the following clinical criteria: hepatomegaly, ascites, elevated bilirubin level, hemodynamic or/and ultrasound evidence of SOS and it should be histologically proven (52, 53). However, these criteria are non-specific and sometimes absent at SOS onset time, causing a delay of diagnosis and treatment. Once again, tissue biopsy for histologic diagnosis might be challenging to obtain in the early post-HCT setting.

Non-invasive ultrasound imaging techniques are used in clinical practice to assist in the exclusion of differential diagnoses, and they can have a role in dubious and in late-onset cases. Nevertheless, the sensitivity and specificity of ultrasound need to be improved for early detection in transplanted patients (54).

In recent years, the incidence of SOS has declined as a result of less aggressive conditioning regimens. The majority of patients with mild-to-moderate disease may resolve SOS spontaneously, whereas the severe forms maintain a high mortality rate (>80%) due to progression to MOF. For this reason, early detection and treatment of SOS is critical for preventing the development of severe SOS and MOF.

The risk of developing SOS is variable (3-15%) and depends upon the type of transplant and the conditioning regimen (49). Allo-HCT recipients have a higher risk of developing SOS compared to auto-HCT recipients (5-15% *vs* 3%, respectively) (36, 51, 55). In addition, patients who receive myeloablative conditioning experience higher rates of SOS (10-15%) compared to those who receive a reduced intensity conditioning (51). Moreover, SOS is more frequent in patients receiving a busulfan‐based conditioning regimens before HCT (38, 56).

The other identified risk factors can be grouped into: 1) factors associated with HCT (e.g. HLA-mismatched donor, second HCT), 2) patient and disease related factors (e.g. older age, female gender, glutathione S-transferase mu 1 polymorphism or C282Y allele), and 3) hepatic related factors (e.g. pre-existing liver diseases, presence of cirrhosis, use of hepatotoxic drugs) (49, 51). Nevertheless, the presence of those risk factors have minimal ability to predict SOS incidence and outcomes (39).

The presence of toxic metabolites derived from conditioning chemotherapies has been proposed as the leading cause of hepatic venule and sinusoidal endothelial cell damage. Drugs breakdown occurs through the hepatic cytochrome P450 complex and produces several toxic side-products which are neutralized by the glutathione enzymatic system (GSH). In patients with a reduced GSH activity toxic side-products accumulate and can damage hepatocytes and sinusoidal endothelium. More specifically, these toxic effects predominantly affect the endothelial cells in the centrilobular veins because this area is poor in GSH. This toxic injury causes a cascade of events that leads to post-sinusoidal hypertension triggering SOS (49).

## Biomarkers of Endothelial Activation and Damage

The endothelium is the first contact for immunological effector cells in the blood and a key regulator in various inflammatory processes. Clinical studies suggested that endothelial dysfunction plays a major role in SOS, GVHD and TA-TMA development. Since EC damage and dysfunction is a major underlying cause of early post-HCT complications, several therapies targeting EC have been investigated. The most promising drugs targeting EC used for prophylaxis and treatment of early HCT complications are: defibrotide, Alpha-1 antitrypsin, statins and acetyl-L-cysteine. A reduced incidence of GVHD, SOS and TA-TMA has been observed with the preventive use of defibrotide (DF), a recognized endothelial protective drug (40), in both adults and paediatric patients (41, 57, 58). Alpha-1 antitrypsin is a serin protease inhibitor that downmodulates inflammation in EC and it has been shown to induce a response in corticosteroid resistant GVHD patients (59). Use of statins and acetyl-L-cysteine, that target EC injury are under investigation in TA-TMA and SOS (60). Moreover, a lower incidence of SOS has been also observed on patients undergoing allo-HCT using statins (61).

The first step toward endothelial dysfunction is loss of vascular integrity and participation of EC in inflammatory response. This change in EC function and morphology could lead to a local increase in permeability, or to major endothelium contraction, resulting into subendothelial exposure (**Figure 1**).

Acute changes in endothelial cell protein expression occur shortly after endothelial activation/injury and alter the protein “landscape” of the cell membrane. These dynamic changes in protein expression can be harnessed as biomarkers or clinical indicators of early endothelial dysfunction. An ideal biomarker for allo-HCT complications should be characterized by a high sensitivity, specificity, and predictive value. Furthermore, it should be cost-effective and reproducible across patient populations. Unfortunately, EC damage biomarkers, which will be discussed in the next section, rarely achieve all the required criteria for the ideal biomarkers, and most of them lack specificity. Nevertheless, this translational biomarker approach could have a clinical role in the next future, as this might enable clinicians to detect alloreactivity at the micro level before it develops into an advanced clinical syndrome. Among those, soluble adhesion molecules, circulating endothelial cells and endothelial cell progenitors, coagulation factors, pro-inflammatory mediators and hyaluronic acid (HA) are all being actively tested (**Table 1**) (33, 62, 66, 109, 110), and several studies are investigating their diagnostic, predictive and prognostic values in early post-HCT complications.

**Table 1:** Main biomarkers (references) described for endothelium damage, and for GVHD, SOS, and TA-TMA prediction, diagnosis, or risk stratification.

Biomakers	Sample requirements	Detection methods	After HCT	Level	GVHD	Level and clinical value	Clinical trial	SOS	Level and clinical value	TA-TMA	Level and clinical value
sICAM-1	Senun/plasma	ELISA	(15, 62–64)	increased	(64, 65)	Increased PG PG		(64–67)	Increased PG Pr		
sVCAM-1	Serum/plasma	ELISA	(62–64)	increased	(64)	Increased PG		(67)	Increased PG D	(64)	Increased PG
sE-selectin	Serum/plasma	ELISA	(62, 64, 68)	increased	(65)	Increased PG		(65)	Increased PG Pr	(64)	Increased PG
sP-selectin	Serum/plasma	ELISA	(62, 64, 68)	increased				(64, 68)	Increased PG Pr		
VWF	Serum/plasma	ELISA	(62, 69)	increased	(65, 69)	Increased		(65, 69)	Increased PG	(70)	increased
TM	Serum/plasma	ELISA	(62, 71)	increased	(65, 69)	Increased		(65, 69	Increased PG	(64, 70)	increased
PAI-l	Serum/plasma	ELISA	(62, 71)	increased				(15, 69, 72, 73)	Increased D PG	(49)	increased
ADAM13	Serum/plasma	ELISA	(15)	increased							
VEGF	Serum/plasma	ELISA	(74)	increased	(75)	Increased PG		(74)	increased		
ANG2	Serum/plasma	ELISA	(62, 63, 71)	increased	(63)	Increased PG		(66)	Increased D PG		
CEC	PB	Flow-cytometry	(76, 77)	increased	(78, 79)	Increased D PG					
EPC	PB	Flow-cytometry	(80, 81)	decreased							
EV	Serum/plasma	Flow-cytometry	(82)	Increased	(83–85)	Antigen-depend. Pg Pr		(66, 86)	Antigen-depend. D PG	
miRNAs	Serum/plasma	RT-PCR, micro-seq. micro-array			(85, 87–92)	miRNA-depend. PG Pr		(82)	Mouse model miRNA-depend. PG		
TNFRI	Serum/plasma	ELISA			(93, 94)	Increased PG Pr	NCT0280694				
TNFa	Serum/plasma	ELISA			(95)	Increased PG Pr		(95)	Increased PG		
ST2	Serum/plasma	ELISA			(93, 96–101)	Increased D PG Pr	NCT0280694	(66)	Increased D		
REG3a	Serum/plasma	ELISA			(93, 98–100)	Increased D PG	NCT0022487	(67)	Increased PG		
TIM3	Serum/plasma	ELISA			(94, 101)	Increased D PG		(67)	Increased PG		
HA	Serum/plasma	ELISA						(66)	Increased D PG		
L-Ficolin	Serum/plasma	ELISA				Increased PG Pr		(66)	Increased D		
IL6	Serum/plasma	ELISA			(94, 102, 103)	Increased D PG		(102, 103)			
sIL2Ra	Serum/plasma	ELISA			(68, 98)	Increased D PG	NCT0022487	(95)	Increased PG		
Haptoglobin	Serum/plasma	ELISA								(104)	Increased PG
NETs	Serum/plasma	ELISA	(40)	increased						(40)	Increased PG
EASIX	panel	panel	(105, 106)	Increased Pr	(86, 107)	Increased PG Pr		(61)	Increased D	(105)	Increased PG
MAGIC	panel	panel			(98–100, 108)	Increased PG Pr					

HCT, stem cell transplant; GVHD, acute graft-versus-host disease; SOS, sinusoidal obstruction syndrome; TA-TMA, transfusion associated thrombotic microangiopathy; miRNAs, microRNAs; EV, extracellular vesicles; CECs, circulating endothelial cells; EPC, endothelial progenitor cells; sIL2Rα, soluble interleukin-2 receptor alpha-chain; ST2, soluble suppressor of tumorigenicity 2; IL6, interleukin 6; TNFR1, tumor necrosis factor receptor 1; REG3α, regenerating islet-derived protein 3α; sICAM-1, soluble Intercellular CAM protein 1; sVCAM-1, soluble vascular CAM protein 1; VWF, Von Willebrand factor; TM, thrombomodulin; HA, hyaluronic acid; PAI-1, plasminogen activator type-1; VEGF, vascular endothelial growth factor; ANG2, Angiopoietin2; NETs, neutrophil extracellular traps; EASIX, Endothelial Activation and stress index panel; MAGIC, Mount Sinai Acute GVHD International Consortium panel; TIM3, T-cell immunoglobulin and mucin domain-containing protein 3; TNFa, tumor necrosis factor alpha; PB, peripheral blood; D, diagnostic; Pr, predictive; PG, prognostic; PB, peripheral blood; ELISA, Enzyme-linked immunosorbent assay; micro-seq, miRNAs sequencing.

### Soluble Biomarkers

Coagulation factors (such as Von Willebrand factor (VWF), thrombomodulin (TM), plasminogen activator type-1 (PAI-1), and VWF-cleaving protease or ADAM13 activity) and soluble cellular adhesion molecules (sCAMs) are reliable markers of EC activation and damage.

Expression of cellular adhesion molecules (CAMs) on EC surface is important for leucocytes adhesion and transmigration through blood vessel into tissues. There are two isoforms of CAMs: circulating soluble CAMs which are either secreted or cleaved, and membrane bound CAMs (111). Several studies analysed coagulation factors and soluble CAMs before and after HCT. VWF, TM and Angiogenic factors (Angiopoietin2 or ANG2), expressed by EC, were found to be increased pre-HCT, compared to healthy controls, suggesting that endothelium is already damaged and activated by the underlying hematologic disease (such as Multiple Myeloma, Lymphoma ore Leukemia) (62, 63).

Moreover, in the first weeks after auto- and allo-HCT an increase of VWF and soluble Intercellular CAM protein 1 (sICAM-1) has been reported (15, 62), while PAI-1, TM and ANG2 are only elevated in patients undergoing allo-HCT (62, 71). In addition, the serum levels of all sCAMs are known to be higher after allo- compared with auto-HCT patients (61).

These data confirm that the microenvironment of the endothelium is significantly altered in patients by the underlying hematologic disease and HCT procedure. Furthermore, additional studies have expanded on these data and discovered changes in soluble protein expression that are specific to patients with post-HCT complications such as SOS, GVHD and TA-TMA.

In another study conducted in patient undergoing allo-HCT (112), serum level of sCAMs and PAI-1 has been measured to evaluate the endothelial dysfunction level after treating patients with recombinant TM, an anticoagulant agent used for transplanted-associated coagulopathy. This study showed that there is an increase of sCAMs and PAI-1 level in all patients, confirming that endothelial damage is a common feature in allo-HCT, but this increase is statistically significant only in patients not treated with recombinant TM. Moreover, patients treated with recombinant TM not only exhibited less endothelial dysfunction level, but also a significant decreased in acute GVHD frequency (112). This study represents a clear example of how endothelial biomarkers could be used to monitor therapy response in patients.

Allo-HCT patients who developed SOS, TA-TMA and GVHD had a significant increase in both VWF and TM levels (65, 69, 70). Furthermore, levels of VWF, and TM (combined with ICAM-1 and E-selectin level) measured one week after HCT were used as SOS predicting biomarker in patients treated with both tacrolimus and sirolimus as GVHD prophylaxis (65).

Circulating levels of PAI-1 were elevated in patients with SOS, but not in those with GVHD (15, 69). Increased PAI-1 levels not only allow differential diagnosis between SOS and GVHD, but also from other liver injuries (72, 73). Conversely, decreased PAI-1 levels within the first two weeks of DF administration has been correlated with complete SOS response at three months post-HCT (57). Nevertheless, PAI-1 marker performed poorly in proteomic-based approach and it was not included as predictive marker in the final composite biomarker panel (66).

Thus, concentration of soluble ICAM-1 and E-selectin is elevated in both GVHD and SOS patients (65); soluble vascular CAM protein 1 (sVCAM-1) increased especially in patients who later develop GVHD and TA-TMA, while P-selectin levels was selectively higher in patients developing severe SOS (64, 68).

Recently, Akil et al. showed that a composite diagnostic biomarker panel (L-Ficolin, hyaluronic acid (HA), VCAM-1) can identify patients at high risk of SOS as early as the first day after HCT, even before clinical manifestation of SOS (66). In addition, the use of the following biomarker panel: suppression of tumorigenicity-2 (ST2), ANG2, L-Ficolin, HA, VCAM-1 was suggested to be useful for SOS diagnosis (66).

Circulating angiogenic factors level (e.g. vascular endothelial growth factor (VEGF) and ANG2) is varied among patients with GVHD and SOS. In some studies, HCT patients had lower VEGF serum concentration compared to healthy volunteers. In contrast, in the same setting, VEGF tends to increase in patients with GVHD and SOS (74, 75). The high level of VEGF might be diagnostic of GVHD, but not predictive as levels of VEGF were not increased prior to GVHD onset (75). In contrast, ANG2 expression is elevated prior to HCT, and there is an additional observed increase in ANG2 levels associated after the development of GVHD (63).

Inflammatory cytokines such as IL2, IL6, IL33, IFNγ, and TNFα are crucial for EC activation and damage (102, 103). TNFα is an inflammatory cytokine involved in damage initiation and spreading in acute GVHD (113). However, TNFα raised also in other post-HCT complications, such as SOS (114, 115). IL2 is a cytokine with critical effects on T-cell physiology. Many studies reported increased soluble IL2 receptor α (sIL2Rα) concentration prior to clinical onset of GVHD and its predictive role in both GVHD development and severity (116–118). Similarly to TNFα, sIL2Rα also rise in other HCT complications, such as SOS and bacterial infections and cannot be considered as a specific biomarker for GVHD (95).

IL33 is a cytokine belonging to IL1 family that binds ST2. Dysregulation of ST2/IL33 signaling pathway was originally described in the context of different inflammatory diseases (119, 120). Additionally, IL33 upregulates the expression of CAMs in human EC (120) and an altered secretion of soluble ST2 by intestinal cells has been observed in experimental models of GVHD (121). Soluble ST2 has been validated as a biomarker for treatment-resistant GVHD, and elevated circulating ST2 at day +7 or +14 post-HCT can also be predictive of NRM following HCT (96, 97).

Discovered as GVHD biomarker by proteomic analysis, ST2 has been correlated with other promising GVHD biomarkers, such as the antimicrobial peptide regenerating islet-derived protein 3 alpha (REG3α), and soluble tumor necrosis factor receptor 1 (TNFR1). These biomarkers have been tested in large multicentre consortia, and identified patients developing GVHD one week after HCT or at disease onset (93, 97, 98).

IL-2Rα, combined with measurement of regenerating islet-derived protein 3 alpha or REG3α [a GVHD target organ-specific damage biomarkers (122)] and ST2 enabled the development of a validated predictive algorithm (Mount Sinai Acute GVHD International Consortium or MAGIC), based on ST2 and REG3α concentrations after one week of systemic glucocorticoid treatment, to predict life-threatening GVHD and NRM (98). Recent studies suggest that the measurement of REG3α and ST2 in haplo HCT may be associated with a higher incidence of GVHD and NRM (99, 100). Moreover, MAGIC predictive algorithm not only is prognostic for NMR and overall survival, but it can be used as response biomarker for acute GVHD treatment (108).

Other biomarker combinations, such as ST2+REG3α+TNFR1 (96), ST2+TNFR1, T cell immunoglobulin and mucin domain-containing protein 3 (TIM3) +TNFR1+IL6 (94), ST2+TIM3 (101), have been investigated in the plasma of HSC patients and improve detection and severity of GVHD. Furthermore, plasma levels of REG3α, sVCAM1, sICAM1, and TIM3 at two weeks post- HCT were consistently elevated in patients who developed SOS (67).

Moreover, a new biomarker panel called EASIX (or Endothelial Activation and stress index) appeared to predict overall survival, NMR, and risk of poor outcomes in patient undergoing to allo-HCT (105, 106). EASIX score combines three routine laboratory tests that are used as diagnostic parameters of TA-TMA and is calculated using the following formula: (Lactate Dehydrogenase (U/L)) x (Creatinine (mg/dL))/Thrombocytes (10^9^ cells per L). Elevated EASIX score at days 0 after HCT has been showed to predict the risk of mortality in patients with acute GVHD and TA-TMA (86, 107). This easy-to-calculate index can be used by clinicians as a tool for clinical management of decision in patient with GVHD in the reduced conditioning regimen setting, since this index was not predictive in case of myeloablative conditioning regimens (86). Furthermore, this index has been tested in a retrospective cohort study analysis to be an independent predictor of risk of developing SOS (61). EASIX have been also used to monitor EC protective efficacy of ursodeoxycholic acid and statins in transplanted patients. Post-HCT prophylactic use of these two drugs was associated with lower EASIX score, which indicate less endothelial damage, lower SOS incidence, and better clinical outcomes (low NMR and high overall survival) (61).

Recently, proteomics profiling on serum from patients undergoing HCT allows the discovery of a 17 KDa haptoglobin degradation product that was differentially expressed in patients who developed TA-TMA. This non-invasive biomarker showed diagnostic value toward TA-TMA and could potentially allow earlier intervention (104). Furthermore, haptoglobin has been proposed to be used in TA-TMA diagnostic criteria (47).

### Circulating Endothelial Cells and Endothelial Progenitor Cells

There are two categories of circulating cells that have been proposed as biomarker of endothelial damage; they include circulating endothelial cells (CEC) and endothelial progenitor cells (EPC). The presence of CEC and EPC in blood and their level measure vascular health homeostasis, being CEC a recognized biomarkers of ongoing endothelial damage, whereas EPC evaluate vascular repair suitability (80, 123).

Prolonged or unregulated endothelial damage often results in a loss of EC integrity and shedding of mature endothelial cells into the bloodstream (124). CEC are a rare cellular subpopulation in peripheral blood and are usually absent in the blood of healthy individuals. They are characterized by the expression of endothelial markers (VWF, CD31, CD144, and CD146) and the absence of leucocyte markers. Currently, the most used biomarkers for CEC detection is CD146, which can be used to increase specificity in combination with other biomarkers, such as VEGFR2 and CD133 (125). Analysis of CEC count in HCT showed that there is an earlier CEC peak in patients receiving total body radiation compared to patients receiving chemotherapy (76). Furthermore, CEC count is lower in patients receiving reduced-intensity conditioning regimen compared to those receiving myeloablative conditioning regimen (77).

By contrast, EPC are bone marrow–derived cells found in peripheral blood that contribute to angiogenesis and to vascular repair after endothelium damage. EPC are characterized by the expression of CD34, CD133, VEGFR2, VWF, CD117, and CD144; a combination of these markers have been used to identify EPC by flow cytometry (123). Although EPC are present in the bloodstream of healthy individuals, patients undergoing allo HCT have lower EPC counts than healthy volunteers, even before transplantation. Even though the EPC count seemed to recover partially twelve month post-HCT, EPC remain lower compared to healthy controls (81).

CEC are considered more sensitive compared to EPC in detecting EC damage. As CEC could even be used as a diagnostic biomarker of EC damage in the early phases of a disease, many studies have been trying to improve the sensitivity of CEC detection and their diagnostic sensitivity (123).

Almici et al. described a relative CEC count increase in patients developing GVHD compared to those without GVHD, and was associated with engraftment as well (78). Moreover, the CEC level can be treated also as marker of GVHD therapy response, as levels tend to return to basal pre-transplant value in responding patients. CEC count is now considered a dynamic phenomenon because is affected by several factors, such as the conditioning regimen, engraftment, infections and immunosuppressive treatments. Nevertheless, cytofluorimetric enumeration of CEC is still not a standardized procedure since different CEC tagging approaches bring complimentary, but not completely overlapping, results (CEC identified as CD146+CD106+CD45-cells or as CD34+CD45-CD146+cells, by CellSearch system or polychromatic flow-cytometry, respectively) (79).

### MiRNAs and Extracellular Vesicles

Two new categories of biomarkers undergoing active investigation are microRNAs (miRNAs) and extracellular vesicles (EVs).

MiRNAs represent a family of non-coding RNAs of 19–25 nucleotides, that regulate gene expression increasing the degradation or blocking translation of a target messenger RNA (126). MiRNAs are presents in all biological fluids and are released by cells mainly in three different forms: as freely circulating miRNAs, as protein-associated (like argonaute 2 and nucleophosmin 1) or encapsulated in EVs. Freely diffusing miRNAs are mainly released by damaged or death cells, whereas miRNAs encapsulated in EVs are specifically released by cells as messengers. Moreover, miRNAs transported by EVs are protected from RNAse degradation, and thus are more stables and specific than freely circulating miRNAs. Several studies suggested the potential use of miRNAs as biomarker in many human diseases including their role as potential biomarker in in early post-HCT complications like GVHD.

Many free circulating miRNAs are differentially expressed in plasma/serum a diagnosis (pre-transplant), two weeks after HCT and before onset of GVHD, such as: miR155, miR146a, miR19a, miR20a, miR30, miR181, miR150, miR194, miR100 and miR518f (87–91) showing a possible prognostic use in GVHD. Moreover, differential expression of some miRNAs, such as miR19b, miR20a and miR30b, has been linked to improved overall survival (91), indicating a possible predictive role in clinical routine.

Mir155 is a strong example of a miRNA which can potentially be used not only as a biomarker but also as therapeutic target. Serum up-regulation of miR155 has been observed in patients with confirmed GI-GVHD (90) and in effector T cells from a murine GVHD experimental model (127). Furthermore, mice receiving miR155-deficient donor lymphocytes had a lower GVHD incidence and improved survival rate, whereas lethal GVHD developed rapidly in mice with T-cell miR155-overexpressions. Consistently, blocking miR155 function with a synthetic oligonucleotide complementary to miR155 improved GVHD symptoms (127).

Clinical studies investigating the role of miRNA as SOS and TA-TMA biomarkers in patients are missing. In a rodent model, where SOS is induced by monocrotaline, serum miRNA profile reveals that miR21-5p and miR511-3p could be used as early predictive biomarkers for SOS (82). Those miRNAs were increased in serum during the early phase of SOS, probably in response to liver sinusoidal EC damage, while the miR122-5p, miR192-5p, and miR101b-3p could be used as indicators for hepatocyte damage in later phase (82). Future clinical studies are necessary to elucidate whether these miRNAs could also be used as biomarkers in TA-TMA and SOS clinical prediction and diagnosis in patients; nevertheless, those preliminary results are encouraging.

Extracellular vesicles (EVs) are cell-derived particles delimited by a membrane, which are important for intercellular communication by shuttling bio-acting molecules (miRNAs, messenger RNA, DNA, proteins, lipids and carbohydrates) through biological barriers from one cell to another (128).

EVs are an highly heterogeneous group of particles including microvesicles and exosomes, which differ for dimensions and cellular origin (128). Owing to their cargo content (miRNAs, proteins, and other bioactive molecules) and capacity to deliver their cargo to specific cells, EVs are involved in homeostasis maintenance and regulation of physiological functions, but also in different pathological processes (e.g. in cancer, inflammation and autoimmune diseases) (129). EVs cargo composition can differ among EVs subpopulations depending on the originating cell type, therefore this vast heterogeneity could be responsible for different biological effects mediated by EVs subgroups (130–132).

The discovery that EVs concentration and cargo composition is altered in patients compared to healthy people suggested their potential diagnostic value. Thus, several studies have been focused on quantification and characterization of serum/plasma EVs membrane proteins (by flow cytometry), and EVs miRNAs content (by molecular techniques).

Endothelial cells constitutively release extracellular vesicles in a low concentration in the blood stream in physiologic conditions. However, endothelial-EVs (EEVs) release increases after EC activation and injury (133, 134). Typically, EEVs express endothelial markers such as CD146, CD105, CD144, CD54, CD62E, CD31, and VWF (135, 136). Furthermore, pro-inflammatory cytokines, circulating angiogenic factors, CRP and PAI-1 trigger the release of EEVs (92, 137, 138). TNFα (92, 139) is a strong activator of ECs and leads to a dose-dependent release of EEVs (137). EVs released after pro-inflammatory cytokine stimulation are enriched with endothelial markers and specific miRNAs (e.g. miR328-3p, let7d-3p, miR59, miR191, miR423) which are generally absent or expressed in lower concentration in physiologic conditions (92). Moreover, the concentration change of EEVs could be used as marker of endothelial dysfunction. As matter of fact, the number of EVs expressing CD31+/annexin V+ or CD42- CD31+ positively correlates with impaired endothelial function and major adverse cardio-vascular events (83, 140, 141), while the decrease of CD62+ EVs has been proposed as biomarker of severe endothelium damage (80, 142). Concentration change over time of EVs wearing these biomarkers combination could be used to monitor EC damage/dysfunction as other “classical” EC biomarkers.

Therefore, besides the concentration change of EEVs, the content and type of EVs released vary during EC activation (143, 144). Thus, temporal analysis of EEVs membrane protein immune-profile and molecular characteristic could provide clinically useful and actionable information on endothelial status and could predict early post-HCT complications.

Recent studies investigated EVs as biomarkers in SOS and GVHD (66, 86). Piccin et al. observed an early post-HCT increase of CD144+ EVs in plasma specimens of SOS patients, which supports the presence of early EC damage (145). Furthermore, the concentration of PAI-1 showed an interesting inverse correlation with EEVs (CD144+) and with EV CD31+/CD41+ levels. This high level of CD144+ EVs observed in the first week post-HCT could have a diagnostic and prognostic role in SOS.

Lia et al. investigated the potential role of serum EEVs as biomarkers of GVHD (84). In this study, they observed a statistically significant expression change post-HCT of three EVs membrane antigens prior to the onset of GVHD, notably an expression increase with CD146, while an expression decrease with CD31 and CD140a. This result showed that monitoring the expression level change compared to basal pre-HCT level of those EVs biomarker could have a prognostic GVHD application in clinical routine. A similar study published by Lia et al. confirmed the correlation between GVHD onset with CD146, CD31 and CD140a plasma EVs expression (85). Furthermore, Zhang et al. has demonstrated that EEVs originated after *in vitro* TNFα stimulation of human umbilical vein endothelial cells are enriched in miR155 (146). Moreover, levels of EVs encapsulated miR155 was significantly higher than free circulating miR155 in both GVHD patients and animal models. The role of miR155 in pathogenesis of GVHD was also confirmed by another study which observed a change in expression of miR155 in serum EEVs before GVHD onset, together with miR100 and miR194 (85). These two studies suggested a possible prognostic use of these miRNAs. Furthermore, inhibition of miR155 by loading a small complementary synthetic RNA molecule inside EEVs reduce differentiation toward proinflammatory T-cell subsets promoting anti-inflammatory response, which ameliorated GVHD effects.

However, despite their promising potential application in the clinical field, EVs and miRNAs use as biomarkers is still in its early stages, mainly due to the lack of standardized protocols in specimen handling, isolation, and analysis methods. Nevertheless, miRNAs and EVs meet the accessibility, high specificity, and sensibility criteria for being an ideal biomarker and the improvement of sampling and isolation techniques, as well as the quantification methods may result in their use as reliable tool in the future.

## Conclusions and Perspectives

The use of non-invasive biomarkers for detection and diagnosis of early post-HCT endothelial complications is a promising field of research with lifesaving implications. There are a number of potential biomarkers that have yet to be validated in clinical trials and a number of studies have also produced conflicting results. The lack of consistency among studies are probably due to differences in technique and treatment, as well as heterogeneity of patients. Reconciling these studies is also difficult due to the differences in timing, intensity of conditioning therapy, source of hematopoietic cells, and applied statistical methods. Thus, predictive biomarkers research might need a multicenter approach with coordinated times of sampling and centralized analysis of biomarker levels and standardized protocols. Moreover, the reduction of confounding variables among studies can be achieved through rigorous selection, acquisition, and storage of biological specimens.

Many of the current existing EC damage/dysfunction biomarkers lack specificity (such as CAMs, inflammatory cytokines, miRNAs, EVs) as change in their levels could be caused by different post-HCT underlying conditions. Nevertheless, they could have some clinical applications either in monitoring response in patients, or in detecting EC damage before its clinical development.

However, differently from other clinical routine tests, these biomarkers need to be tested at multiple time points and the comparison of pretransplant levels with later ones is essential to predict outcome.

At the moment, among the proposed GVHD biomarker panels, MAGIC is the closest to clinical application, as it has been validated and some clinical centers have recently started to use it. By contrast, available data on biomarkers for SOS and TA-TMA close to clinical use are still missing, and more studies are needed to identify more reliable and useful markers. Nevertheless, recently EASIX panel showed its value in stratifying patients at high risk for SOS, and in predicting OS and NRM in reduced intensity conditioning regimen allo-HCT. It is predictable that this panel, which is composed by validated routine clinical tests, will come into use in allo-HCT setting in the next future.

The Proteomics approaches has promoted the discovery or more sensitive and specific biomarker, which composed the validated MAGIC panel.

In the next future, advances in the field of Omics methodologies will expand the library of new biomarkers following a biology-driven development approach. Biology driven approaches for identification of combinatorial biomarker panel are generally divided in three distinct phases characterized by different technology approaches: Biomarker Discovery, Validation and Qualification. The Omic- approaches (e.g. mass spectrometry based proteomic approaches, array based transcriptomes approaches), generating large amount of data, are suitable for biomarker discovery and the number of potential generated biomarker candidates need to be refined using bioinformatics tool. The discovered biomarkers (usually up to 10-100 candidates) need then to be validated in other independent cohort using different reliable techniques (like ELISA). Data obtained are then analysed using data mining methods that are based on biological information’s such as tissue expression patterns, clinical relevance, pathways analysis tool, etc. The validated biomarkers (generally <10) need to be tested in the qualification phase on clinical trial.

Biology driven approaches in biomarker development have the disadvantages to be time consuming, but these approaches could potentially find more sensitive and specific biomarkers.

No single biomarker presented in this review is sufficiently sensitive or specific by itself as either a diagnostic or predictive/prognostic test. Due to the involvement of many factors promoting EC damage and pathogenesis of these complications, a panel of biomarkers has more chance to turn into a prognostic or diagnostic tool in the next future.

## Author Contributions

GL, LG, SL and BB devised and wrote the manuscript. All authors contributed to the article and approved the submitted version.

## Funding

This work was supported by Italian Ministry of Education, University and Research (MIUR Bando Ricerca Locale 2018 and 2019).

## Conflict of Interest

The authors declare that the research was conducted in the absence of any commercial or financial relationships that could be construed as a potential conflict of interest.
